# How Droplets Can Accelerate Reactions—Coacervate Protocells as Catalytic Microcompartments

**DOI:** 10.1021/acs.accounts.4c00114

**Published:** 2024-07-05

**Authors:** Iris B. A. Smokers, Brent S. Visser, Annemiek D. Slootbeek, Wilhelm T. S. Huck, Evan Spruijt

**Affiliations:** Institute for Molecules and Materials, Radboud University, Heyendaalseweg 135, 6523 AJ Nijmegen, The Netherlands

## Abstract

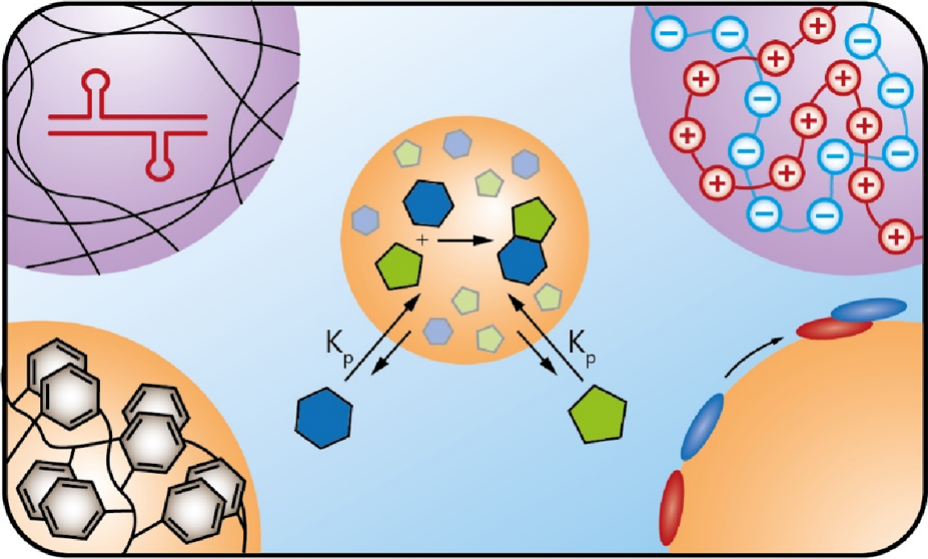

Coacervates are droplets formed by liquid–liquid phase separation (LLPS) and are often used as model protocells–primitive cell-like compartments that could have aided the emergence of life. Their continued presence as membraneless organelles in modern cells gives further credit to their relevance. The local physicochemical environment inside coacervates is distinctly different from the surrounding dilute solution and offers an interesting microenvironment for prebiotic reactions. Coacervates can selectively take up reactants and enhance their effective concentration, stabilize products, destabilize reactants and lower transition states, and can therefore play a similar role as micellar catalysts in providing rate enhancement and selectivity in reaction outcome. Rate enhancement and selectivity must have been essential for the origins of life by enabling chemical reactions to occur at appreciable rates and overcoming competition from hydrolysis.

In this Accounts, we dissect the mechanisms by which coacervate protocells can accelerate reactions and provide selectivity. These mechanisms can similarly be exploited by membraneless organelles to control cellular processes. First, coacervates can affect the local concentration of reactants and accelerate reactions by copartitioning of reactants or exclusion of a product or inhibitor. Second, the local environment inside the coacervate can change the energy landscape for reactions taking place inside the droplets. The coacervate is more apolar than the surrounding solution and often rich in charged moieties, which can affect the stability of reactants, transition states and products. The crowded nature of the droplets can favor complexation of large molecules such as ribozymes. Their locally different proton and water activity can facilitate reactions involving a (de)protonation step, condensation reactions and reactions that are sensitive to hydrolysis. Not only the coacervate core, but also the surface can accelerate reactions and provides an interesting site for chemical reactions with gradients in pH, water activity and charge. The coacervate is often rich in catalytic amino acids and can localize catalysts like divalent metal ions, leading to further rate enhancement inside the droplets. Lastly, these coacervate properties can favor certain reaction pathways, and thereby give selectivity over the reaction outcome.

These mechanisms are further illustrated with a case study on ribozyme reactions inside coacervates, for which there is a fine balance between concentration and reactivity that can be tuned by the coacervate composition. Furthermore, coacervates can both catalyze ribozyme reactions and provide product selectivity, demonstrating that coacervates could have functioned as enzyme-like catalytic microcompartments at the origins of life.

## Key References

- AbbasM.; LipińskiW. P.; NakashimaK. K.; HuckW. T. S.; SpruijtE.A Short Peptide Synthon for Liquid–Liquid Phase Separation. Nat. Chem.2021, 13 ( (11), ), 1046–105434645986 10.1038/s41557-021-00788-x.^1^*A short peptide synthon consisting of two hydrophobic dipeptides coupled by a linker with a large dipole moment can form coacervate droplets. Inside these droplets, hydrophobic guests are enriched and reactions are accelerated 40- to 300-fold*.
- SmokersI. B. A.; van HarenM. H. I.; LuT.; SpruijtE.Complex Coacervation and Compartmentalized Conversion of Prebiotically Relevant Metabolites. ChemSystemsChem.2022, e202200004.^2^*Prebiotic metabolites with a minimum of three “interaction sites”—either a negative charge or aromatic group—can form coacervates with R*_10_. *Inside metabolite coacervates, the oxidation of NADH by ferricyanide is accelerated*.
- WangJ.; AbbasM.; WangJ.; SpruijtE.Selective Amide Bond Formation in Redox-Active Coacervate Protocells. Nat. Commun.2023, 14 ( (1), ), 1–1138129391 10.1038/s41467-023-44284-xPMC10739716.^3^*Peptide bond formation through amino thioacid oxidation in pLys/ferricyanide coacervates is enhanced inside the droplets and shows selectivity for amino acids that interact less strongly with the coacervate matrix*.
- LipińskiW. P.; VisserB. S.; RobuI.; FakhreeM. A. A.; LindhoudS.; ClaessensM. M. A. E.; SpruijtE.Biomolecular Condensates Can Both Accelerate and Suppress Aggregation of α-Synuclein. Sci. Adv.2022, 8 ( (48), ), 649510.1126/sciadv.abq6495PMC1094278936459561.^4^*The coacervate interface can catalyze reactions: The Parkinson-related protein alpha-Synuclein can localize at the interface due to its amphiphilic nature, which accelerates aggregation of the protein*.

## 1 Introduction

Catalysis is the acceleration of chemical reactions by molecules or structures that are themselves not consumed by the reaction. Catalysis plays a central role in life, with enzymes being the main driver of cellular reactions. The emergence of catalysis must have been an essential step at the origins of life. Considering the presumably dilute concentrations of organic molecules on the early Earth, catalysis would have played a crucial role in enabling chemical reactions to occur at appreciable rates and overcoming competition from hydrolysis. Catalysts can also provide selectivity, allowing for accumulation of certain molecules in the prebiotic soup and control over the reactions that take place. In the RNA-world scenario, catalysis by ribozymes therefore plays an essential role.^5^

In addition to RNA catalysts, other simple molecules and assemblies may have catalyzed reactions. Many of these, including transition metals^6,7^ and peptides,^8−10^ are catalysts in the classical sense: they lower the activation energy of a reaction without affecting the overall change in Gibbs free energy. Chemical reactions can, however, be enhanced in multiple other ways that have a similar effect: faster conversion, more product and higher selectivity. In particular, the physical environment in which a reaction takes place can lead to effective concentration enhancement, stabilization of products, destabilization of reactants, lowering of the transition state and selectivity in product outcome, and can therefore substitute a catalyst. Examples of such environments include paste or wet/dry cycles,^11,12^ eutectic ice phases,^10,13^ concentration at surfaces of minerals^14^ and thermal gradients in rock pores.^15^ Interestingly, protocellular compartments such as vesicles^16^ and coacervate droplets^2,3,17−23^ can locally provide a different environment, allowing them to act as catalytic microcompartments similar to micellar catalysts,^24^ and underlining their importance even at the early stages of the origins of life. In this Accounts, we focus on coacervates and discuss in detail the mechanisms by which they can accelerate prebiotic reactions, and complement this with experimental examples.

## 2 Coacervates as Protocellular Compartments

Coacervates are droplets formed by liquid–liquid phase separation (LLPS), resulting in two aqueous phases: a solute-rich dense phase dispersed in a dilute supernatant phase. LLPS is driven by multiple weak associative interactions, such as charge–charge, cation-π and π–π interactions, or by the hydrophobic effect.^25^ If these interactions occur between different parts of the same molecule, the resulting droplets are formed by a single species, and are called simple coacervates. If these interactions occur between two different molecules, the resulting coacervates are formed by multiple species and are called complex coacervates. These droplets were first proposed as protocells by Oparin in 1936.^26^ Protocells are cell-like compartments that are hypothesized to have preceded the first living entities, but to have been essential in their emergence by compartmentalizing prebiotic reactions. Coacervate protocells recently gained renewed interest due to the discovery that modern cells contain membraneless organelles (MLOs) that are formed by LLPS, such as the nucleolus and stress granules.^27^ Their continued presence in cells could point at a possible prebiotic origin of MLOs and a role for phase separated compartments even in the early stages of the evolution of life.

## 3 Prebiotically Plausible Coacervates

Since their discovery, coacervates have mostly been made by large macromolecules such as proteins, nucleic acids or synthetic polymers, which were likely not available on the early Earth. It has recently been shown that also small and prebiotically plausible molecules, such as short peptides and small multiply charged molecules, can form coacervates. Although a critical length and charge density are required to have sufficiently strong interaction between the oppositely charged components to overcome the mixing entropy,^25,28^ this length seems to be within reach of prebiotic chemistry.^29^ Charged homopeptides as short as 5–10 amino acids undergo LLPS, either with an oppositely charged peptide,^30^ with RNA^31^ or with smaller charged molecules. We have shown that prebiotic metabolites with a minimum of three “interaction sites”—either a negative charge or aromatic group—can undergo LLPS with R_10_.^2^ This includes nucleotides, intermediates of prebiotic TCA cycle analogues, inorganic phosphates as short as pyrophosphate, and commonly used prebiotic reagents such as trimetaphosphate, NADH and ferricyanide. The prebiotic availability of arginine and lysine is sometimes debated, and it is suggested that they were preceded by 2,3-diaminopropionic acid and ornithine.^9^ Interestingly, short peptides in which arginine was replaced by ornithine were still able to form complex coacervates with RNA.^32^ Noteworthy is that peptides are not strictly required for coacervate formation. Even divalent metal ions such as Mg^2+^ and Mn^2+^ can be used as a cation and can form coacervates with RNA^33^ and polyphosphate.^34^

In addition to complex coacervates, hydrophobic simple coacervates might have existed on the early Earth. Several short peptides and peptide derivatives have been reported that form simple coacervates, including histidine-rich squid beak proteins,^35^ mussel-foot protein derivatives^36^ and designer peptides enriched in arginine and aromatic amino acids.^37^ Our group showed that even smaller peptide derivatives containing two hydrophobic/aromatic “sticker” dipeptides joined by a linker with a large dipole moment can form coacervate droplets.^1^ And more recently, even single dipeptide esters were found to undergo LLPS.^38^ In addition to peptides, also other hydrophobic molecules such as fatty acids can form simple coacervates.^39^

## 4 Effects on Chemical Reactions: Rate Acceleration and Selectivity through Compartmentalization

Both simple and complex coacervates have been shown to accelerate a wide range of reactions: enzymatic^40−44^ and nanoparticle-catalyzed^40^ reactions, cell-free gene expression^45^ and reactions between synthetic small molecules,^1,38,46^ as well as prebiotically relevant reactions such as the oxidation of NADH by ferricyanide,^2^ peptide bond formation through amino thioacid oxidation,^3^ DNA ligation^18^ and ribozyme reactions such as Hammerhead and hairpin ribozyme catalysis,^19,23^ catalyzed RNA ligation^20,21^ and Azoarcus ribozyme self-replication.^22^

In this section, we discuss in detail the properties of coacervates that can cause this rate enhancement. As mentioned in the introduction, coacervates can affect the rate and selectivity of reactions in a manner similar to micellar catalysts.^24^ For any reaction, the rate is composed of a rate constant and the concentration(s) of reagent(s), e.g. for a second order reaction A + B → P the rate is given by . Coacervates could affect both parts, as they could (i) locally increase the concentration (or activity) of reagents and/or (ii) lower the effective energy barrier for the reaction by changing the energy landscape. In the next part, we will elaborate on these factors and discuss how they can lead to reaction rate enhancement.

### 4.1 Local Reagent Concentration

Coacervates spontaneously localize a wide range of molecules if they have a favorable interaction with the coacervate material, based on the same types of interaction that drive phase separation (Section 2). The coacervate interior is more hydrophobic than the surrounding solution, and can be rich in charged and/or aromatic species. Hydrophobic, aromatic and (multiply-)charged molecules therefore preferentially accumulate inside the coacervate droplets, as do molecules that form base-pairs with the coacervate components (Section 4.3).^47^ Molecules that do not have favorable interaction with the coacervate material will preferentially remain in the dilute phase. The uptake of guest molecules in a coacervate is described by the ratio of concentrations in the two phases or partition coefficient . A wide range of biologically and prebiotically relevant molecules has been shown to have an increased concentration inside coacervates, including RNA,^48−50^ proteins,^39,40^ peptides,^49,50^ nucleotides,^48^ phospholipids,^31^ divalent metal ions^17,48,50,51^ and prebiotic reagents such as ferricyanide^2,3^ and NADH.^2^ The concentration effect can be as strong as 10,000-fold,^48^ but is often in the range of 2- to 50-fold.^1,2^ In addition to accumulating in the coacervate interior, molecules can localize to the interface of coacervates. We will discuss this effect in Section 4.2.5.

**Figure 1:**
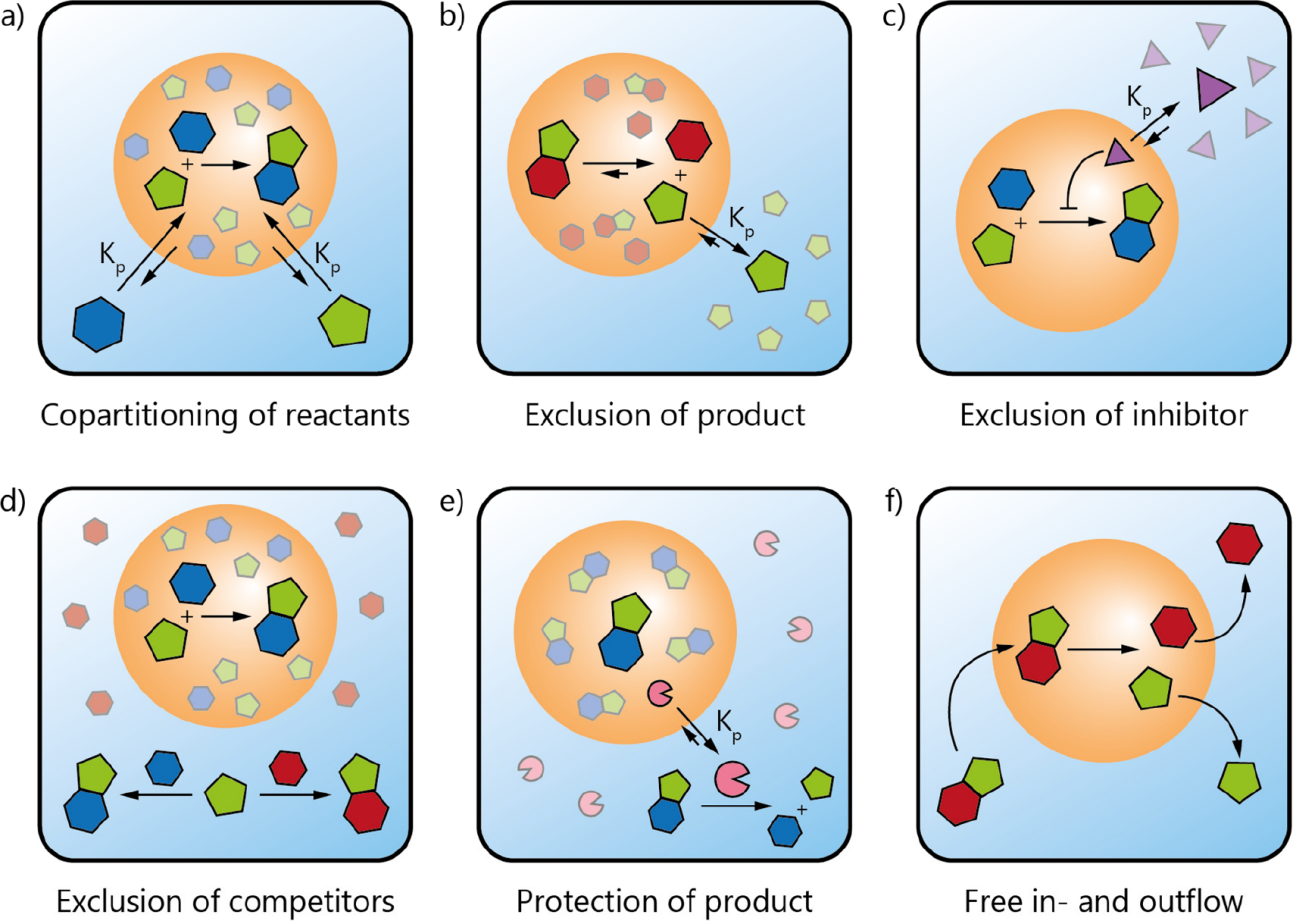
Enhancement of reaction rates, product accumulation and reaction selectivity by local concentration in coacervates. (a) Copartitioning of reactants leads to faster reaction rates inside the coacervate. (b) Exclusion of the product of an equilibrium reaction can shift the equilibrium toward product formation. (c) Exclusion of an inhibitor can relieve reaction inhibition and thus lead to acceleration. (d) Exclusion of a competing reagent from the coacervate can give product selectivity. (e) Protection of labile products by exclusion of molecules that break them down. (f) To maintain the partition coefficients during a reaction inside the coacervate, reagents are continuously supplied from the dilute phase, while product diffuses out.

The increased local concentration could accelerate reactions when reagents copartition into the coacervate (Figure 1.a). Most of the experimental examples of reaction rate enhancement inside coacervates do, in fact, show that the reagents accumulate in the coacervate.^1,2,22,40−44,46^ Locally concentrating reagents in coacervates could have also been essential in avoiding dilution of prebiotic reactions. As we will discuss in Section 4.2.4, dilution unfavorably shifts the balance between formation of complex molecules such as RNA and their hydrolysis, troubling information storage in prebiotic systems.^52^

It should be noted, however, that from a thermodynamic perspective reaction rates are determined by the reagent’s activity rather than concentration. Bauermann et al. showed that in systems at phase equilibrium, this implies that reaction rate differences always stem from differences in reaction rate constants (which we discuss in Section 4.2), as chemical potentials and, hence, activities are equal between different phases.^53^ In other words, a high local concentration of reactants due to strong partitioning is coupled to a low activity coefficient with inverse proportionality. However, it remains to be seen if phase equilibrium is maintained in experimental systems of coacervates hosting chemical reactions, or if (mass) transport limitations and slow coacervate relaxation mean that chemically reactive coacervates should be considered out-of-equilibrium systems. Moreover, in the case of diffusion-limited reactions local concentrations would have had direct influence on the encounter probability and reaction rate.

A second mechanism through which reaction rates could be enhanced by partitioning is by exclusion of a product or inhibitor from the coacervate. Reaction equilibria can be shifted to favor product formation by exclusion of the product from the condensate (Figure 1.b). This is similar to the mechanism-of-action of phase transfer catalysts. Exclusion of inhibitors can similarly enhance reaction rates (Figure 1.c), while exclusion of competing reagents can help to avoid side reactions (Figure 1.d). In a similar vein, coacervates could protect complex and labile molecules such as RNA from degradation by excluding molecules that break them down (Figure 1.e).

The free exchange of molecules between the coacervate and the dilute phase is an another important factor in the kinetics of reactions in coacervates^53^ and can be used to “drive” reactions: to maintain the partition coefficients, reagents that are consumed in the coacervates are continuously supplied from the dilute phase, while products diffuse out to the dilute phase (Figure 1.f). Beneyton et al. showed that for the enzyme formate dehydrogenase, the free in- and outflow of substrate and product leads to at least a 2-fold increase in rate when compared to the reaction taking place in a pure coacervate phase.^54^

Finally, the coacervate volume affects the rate of reactions that take place inside. Theory and kinetic models show that bimolecular reactions are accelerated most when partitioning is as high as possible, but that for a given *K*_p_, there is an optimal amount of coacervate phase for which the highest rate enhancement is achieved.^55,56^ In addition to the relative amount of coacervate phase, also the size of individual coacervate droplets can influence reactions. When molecules cannot be freely exchanged with the dilute phase due to interfacial resistance,^57^ reaction kinetics become dependent on the total coacervate surface area, and thus on the size and number of droplets.

### 4.2 Effect of Physicochemical Environment on Reaction Energy Landscape

**Figure 2:**
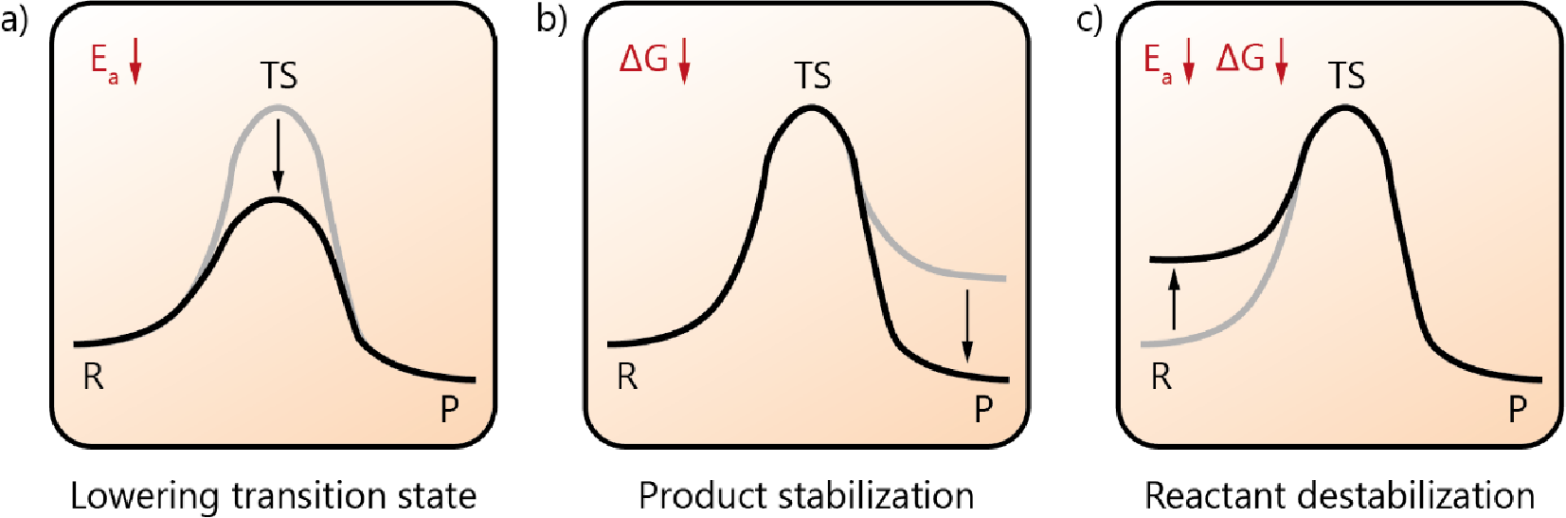
Local physicochemical environment inside the coacervates can significantly change the energy landscape of chemical reactions, affecting both the kinetics and thermodynamics. It can (a) stabilize the transition state and thereby lower the activation energy, (b) stabilize the product and thereby lower the Gibbs free energy, and (c) destabilize the reactant and thereby lower the activation energy and the Gibbs free energy of the reaction.

The second way in which coacervates can alter reaction rates is by changing the energy landscape of the reaction. The physicochemical microenvironment inside the coacervate is substantially different from the surrounding dilute phase, and can stabilize the transition state with respect to the reactants and thereby lower the activation energy, stabilize the product with respect to the reactants, or destabilize the reactants (Figure 2). In these ways, it can accelerate reactions and favor reactions that are not favorable in (dilute) aqueous solution. We have shown that the reaction between a hydrophobic enol and aldehyde hardly occurred in aqueous solution, but was accelerated 40- to 300-fold inside hydrophobic simple coacervates, due to a decrease of the effective energy barrier by 6.3 kJ mol^–1^.^1^ Also work by Koga et al.,^40^ Jacobs et al.,^46^ Peeples and Rosen^41^ and Küffner et al.^44^ has shown experimentally that reactions can be accelerated by properties of coacervates that affect the energy landscape.

In this section, we further discuss the different ways in which the local coacervate environment can affect the reaction rate, including the local polarity and charge density, crowding, effective pH and water activity, and the presence of catalytic molecules. Although some of these properties could be considered concentration effects (of H^+^, water and catalysts) we evaluate them here as bulk properties of the coacervate phase since they are also present in absence of reagents.

#### 4.2.1 Coacervate Polarity and Charge-Density

**Figure 3:**
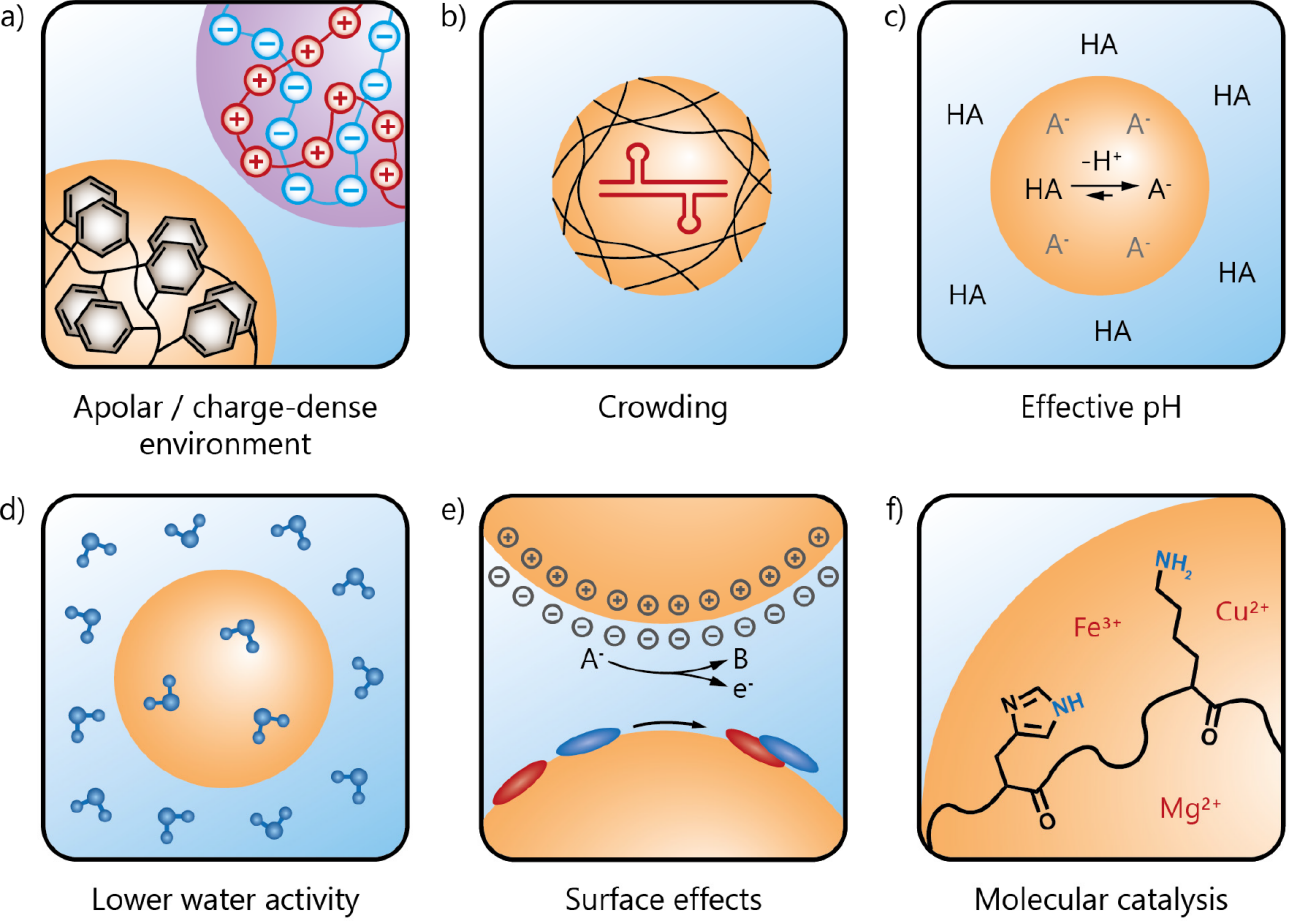
Properties of the coacervate local environment that can alter the energy landscape of reactions. (a) The coacervate phase is apolar and often charge-rich, which can (de)stabilize reactants, transition states and products of reactions. (b) Local crowding and high viscosity can accelerate reactions involving complexation of large molecules such as ribozymes. (c) Coacervates can have a distinct effective local pH from the surrounding solution, which can drive reactions involving (de)protonation. (d) The reduced water activity of coacervates can favor condensation reactions and reactions that are sensitive to hydrolysis. (e) The charged surface of coacervates can accumulate molecules and accelerate their reaction in a similar manner as heterogeneous catalysis. (f) Classical catalysts such as divalent metal ions and catalytic amino acids can be accumulated inside the coacervate and can locally catalyze reactions.

Inside the coacervate, the charged and/or hydrophobic peptides that drive LLPS are condensed (Figure 3.a). This leads to a high local enrichment of charges and relatively apolar peptide backbones. The high concentration of apolar moieties lowers the effective polarity inside the droplets, which is reported to be closer to organic solvents such as DMSO or methanol than to water.^40,44,58^ This is an interesting similarity with enzyme active sites, which form an apolar environment to drive conversion of substrates.

In general, a more apolar environment leads to strengthening of charge–charge interactions, but also to destabilization of transition states involving charge separation. However, complex coacervates accumulate molecules with high charge densities, leading to a local environment with a higher volumetric charge density than the surrounding solution. Such an environment can screen charges more effectively, and stabilize transition states involving separation of charges, in contrast to the apolar environment. The precise balance of these counteracting effects depends on the coacervate composition and type of reaction that is localized.

Destabilization of the reactants in coacervates has been shown for dehybridization of DNA and RNA, an essential step in the (self-)replication of nucleic acids^52^ and the functioning of many ribozymes (Section 5).^20^ Cation-pi interactions between the coacervate components and the nucleobases destabilize the hybridized strands^59^ an effect that is stronger when the coacervates are made from longer peptides^30^ and which is enhanced even further in multiphase coacervates.^49^ Jacobs et al. also showed that a labile reaction product could be accumulated inside coacervates because it was stabilized by the coacervate environment.^46^

The coacervate environment can, however, also stabilize the reactant, leading to deceleration of reactions. Such an effect was observed by our group for the reaction between a hydrophobic enol and aldehyde inside simple hydrophobic coacervates introduced above.^1^ Although partitioning of the reagents was stronger in phenylalanine-rich droplets than in leucine-rich ones, the reaction was faster in the leucine-rich droplets, probably due to too strong stabilization of the reagents in the phenylalanine-rich droplets.

#### 4.2.2 Crowding and Viscosity

A second factor that can affect reaction rates in coacervates is crowding due to the high macromolecular content of the droplets (Figure 3.b). The excluded volume effect generally favors complexed or compact states of macromolecules, and can favor reactions that result in a reduction of the volume of the product compared to the reagents.^60^ For endergonic reactions where the transition state is most similar to the complexed product, the activation energy can be significantly reduced by stabilization of the transition state. Although this effect is probably limited for prebiotic reactions involving small molecules, it can influence ribozyme reactions. Strulson et al. have shown that in a crowded aqueous two-phase system ribozyme activity is enhanced 70-fold.^61^

The high macromolecular content of coacervates does not only give rise to crowding, it also increases the viscosity. Coacervate viscosities are reported to be 10^2^–10^3^-fold higher than water,^44,62^ and this is expected to slow down diffusion-limited reactions. Again, this effect may be limited for prebiotic small molecule reactions, but could be more pronounced for ribozymes and other large molecules.

#### 4.2.3 Effective pH

As explained in Section 4.2.1, the lower polarity and high concentration of charged amino acid residues inside the coacervate can (de)stabilize charges. It is therefore likely that protonation equilibria will be locally shifted, leading to altered p*K*_a_ values and an effective local pH that differs from the pH in the dilute phase (Figure 3.c). A similar effect occurs in enzyme active sites, and this could locally drive reactions that are dependent on a protonation or deprotonation step and could affect acid-/base-catalysis, as was postulated by Jacobs et al. to be one of the main driving forces for the enhanced formation of a synthetic imine inside poly(acrylic acid)-rich coacervates.^46^

Such a shift in protonation equilibrium was observed in measurements with a pH-sensitive SNARF dye, which suggested an effective pH difference of up to 0.6 units between the coacervates and the dilute phase.^30^ It was shown that this apparent difference in pH stems from electrostatic interactions between the SNARF dye and the positively charged amine groups on polyK, leading to a shift in p*K*_a_ of the dye.^63^ Such modulation of the p*K*_a_ of guest molecules is interesting from the perspective of catalysis and chemical reactivity, as this can have a pronounced effect on nucleophilicity, as we discuss below for catalytic lysines (Section 4.2.6). Finally, Testa et al. observed that even larger effective pH differences of up to 2 units could be generated in an out-of-equilibrium system, by compartmentalizing a base-producing reaction: the formation of ammonia by urease.^64^

#### 4.2.4 Lower Water Activity

Many prebiotic reactions are severely hindered by hydrolysis; either because the reagents are hydrolytically labile, for example in the case of activated phosphates^11^ and -nucleotides,^13^ or because the product is not stable, as is the case for RNAs.^13^ These reactions typically require reduced water activity to function, which can be obtained inside pastes or eutectic ice phases.^11−13^ Also condensation reactions like polymerization can greatly benefit from such conditions.^12^ Although the water content of coacervates is only slightly reduced—coacervates are reported to have a water content ranging between 50–90 wt %,^1,40,51,65^ which translates to 97–99 mol %—the high macromolecular content of coacervates could significantly reduce the amount of “free” water that is not bound in a hydration shell, i.e. the local water activity (Figure 3.d).^66^ We therefore hypothesize that inside coacervates the water activity could be reduced enough to facilitate reactions that suffer from competition with hydrolysis in dilute solution.^46^ A similar mechanism is also used by enzymes, which exclude water from their active site to drive conversion of substrates.

#### 4.2.5 Surface Effects

The surface of coacervates is another potential spot for increasing the rate of chemical reactions (Figure 3.e). Surface catalysis of prebiotic reactions at liquid interfaces has been well described, although usually for air–water interfaces in aqueous aerosols or microdroplets. Phosphorylation,^67^ ribonucleoside formation^68^ and peptide bond formation^69^ have all been shown to be promoted by air–water interfaces. The liquid–liquid interface between coacervates and the surrounding dilute phase holds similar promise for surface catalysis.^25,70^

The coacervate interface is typically charged,^62^ allowing for accumulation of oppositely charged molecules at the surface. Additionally, amphiphiles may interact with the hydrophobic coacervate core and accumulate at the interface. One might expect that the surface-limited diffusion of molecules at the interface could lead to higher rates of collisions between molecules due to a reduction in diffusion dimensionality, but the rate of collisions in the bulk or at the interface is roughly equal for equal concentrations.^71^ The surface can, however, increase the reaction rate in a manner similar to heterogeneous catalysis: by concentrating reagents and bringing them together in the right conformation and orientation for reaction.

To illustrate this effect, experimental results show that the formation of amyloid fibrils from peptides and proteins can be accelerated at the interface of coacervates.^4^ One explanation for the enhanced rates is the extended conformation of molecules at the coacervate interface, with an orientation perpendicular to the interface.^72^ Although small prebiotic molecules are not expected to experience such large conformational differences, reactions of larger amphiphilic peptides or polymers might be aided by such a mechanism.

The properties of the bulk coacervate phase that can increase reaction rates, as discussed above, also influence reactions at the interface, with one major difference: at the interface there is typically a gradient in these properties, such as charge, pH and water activity. As mentioned in Section 4.2.3, Testa et al. showed that by compartmentalization of urease into coacervates a stable pH gradient could be formed between the dense and dilute phase by the local production of ammonia.^64^ Fast acid- or base-producing prebiotic reactions could potentially provide a similar gradient. The gradient in charge produces a surface potential that could influence reactions involving charges such as redox reactions, as suggested by Dai et al.^73^ The formation of electrochemical, redox and proton gradients is thought to be essential in providing energy for the emergence of life,^74^ and makes the coacervate interface an exciting reaction environment for prebiotic chemistry.

#### 4.2.6 Metal Ion and Molecular Catalysis

Lastly, metal ion and “molecular” catalysts can be accumulated in coacervates (Figure 3.f). Divalent metal ions such as Mg^2+^^17,48,50^ and Ca^2+^^51^ have been shown to significantly partition into coacervates, and other catalytically active multivalent metal ions such as Fe^2+^, Fe^3+^ and Zn^2+^ are expected to be similarly localized. These metal ions can catalyze a wide range of reactions such as phosphorylation, rTCA cycle conversions and many more.^6,7^ Additionally, Mg^2+^ is essential for the functioning of most ribozymes, as discussed in Section 5.

In addition to metal ions, coacervates can be rich in peptides with specific catalytic activity.^29^ Short histidyl peptides have been shown to catalyze phosphorylation reactions,^8^ RNA oligomerization from activated nucleotides^10^ and the formation of peptide bonds.^16^ Catalytic activity of lysine residues has been shown in self-replicating macrocycle stacks, where several lysine residues are brought in close proximity which lowers their p*K*_a_ by about 3 units and increases nucleophilicity.^75^ Catalytic activity has also been shown for the prebiotically plausible lysine precursor 2,3-diaminopropionic acid, which has a similar p*K*_a_ as the catalytically active lysines in the stacks.^9^ Furthermore, positively charged peptides can enhance ribozyme functionality at low Mg^2+^ concentrations, as we will discuss in Section 5. Lastly, short homochiral peptides have been shown to impose homochirality on reaction products through stereospecific catalysis. Homochiral dipeptides such as l-Val-l-Val have been shown to give up to an 82% enantiomeric excess in the production of D-erythrose from glycolaldehyde^76^ and even higher in nonprebiotic aldol condensation reactions.^77^ The increased local concentration of these catalysts can lead to more effective catalysis inside coacervates.

### 4.3 Selectivity in Reaction Outcomes

In addition to accelerating reactions, the coacervate environment can induce selectivity in reactions. Favoring of one reaction over another can be realized either by differences in partitioning or by affecting the energy landscape of two reactions differently.

Partitioning gives rise to selectivity if different reactants do not partition to the same extent. This could reduce the formation of side products by exclusion of competing reagents (Figure 1.d). Even small differences in molecular structure can give rise to differential partitioning. Our group and several others showed that guest RNAs that are complementary to the coacervate component RNAs are selectively taken up over noncomplementary strands, with up to 50-fold higher partitioning.^48,78^ Also length (and concomitant number of charges) is an important contributor to partitioning, with longer sequences generally showing stronger partitioning. Drobot et al. have shown that 39-mer RNAs have a 3-fold higher partition coefficient than a 12-mer RNA in pLys/CM-dextran coacervates.^23^ Selectivity in uptake based on sequence and length can result in preferential replication of specific and longer self-replicating RNAs, and thereby influence their evolution.^52^

Second, selectivity can be obtained by affecting the rate constant of the reaction. As mentioned in Section 4.2.1, reactants that have a strong interaction with the coacervate matrix will be stabilized, and therefore become less reactive. When different reactants interact differently with the coacervate matrix, this can lead to reaction selectivity based on the difference in interaction strength of the reactants. Our group recently reported an example of this phenomenon: thioacid ligation in pLys/ferricyanide coacervates occurs more readily for glycine than for glutamic acid, with glycine almost completely outcompeting glutamic acid.^3^ Even though both amino acids have comparable nucleophilicities, the charge–charge interaction of glutamic acid with the coacervate material significantly reduces its reactivity, showing that coacervates can induce strong selectivity on reactions.

## 5 Case Study: Ribozyme Reactions in Coacervates

As an illustration of our analysis of how the coacervate microenvironment could influence chemical reactions, we look into ribozyme activity in coacervates. In recent years, there has been increasing focus on reconciling the functional repertoire of ribozymes with the distinct microenvironment of coacervate compartments. This has resulted in a range of systems that not only displayed ribozyme activity inside coacervates, but also provided a better understanding of the role of coacervate protocells in steering prebiotic chemical reactions.

### 5.1 Functional Scope of Ribozymes in Coacervates

**Figure 4:**
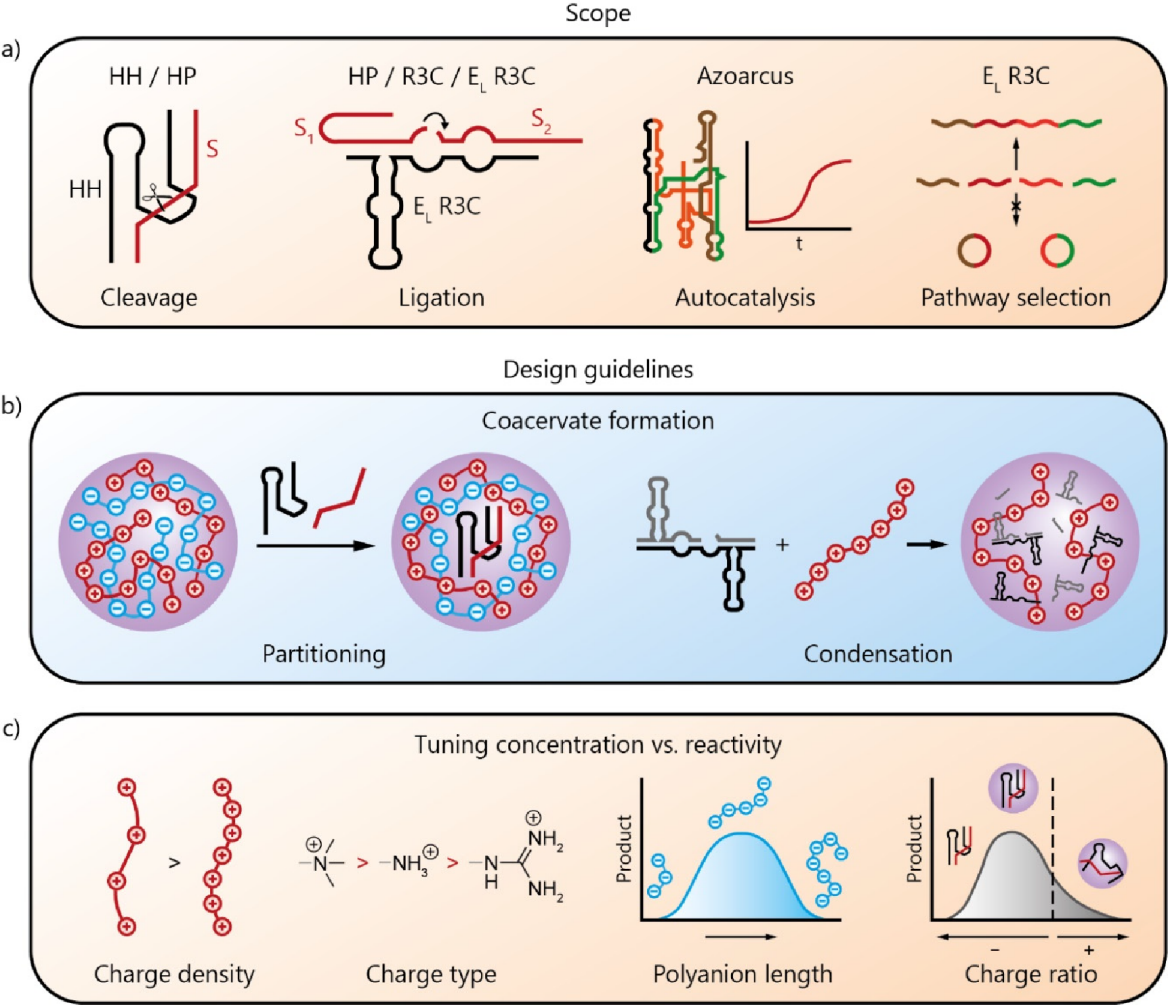
Ribozyme reactions inside coacervates. (a) Scope of ribozyme reactions that can take place in coacervates.^19,21−23^ (b, c) Coacervate design guidelines for optimal functioning of ribozyme reactions. (b) Ribozymes can be partitioned into preformed coacervates or the ribozymes can be used as a coacervate component through condensation with a polycation.^19,50^ (c) To tune the local concentration of ribozyme versus its reactivity inside the coacervates, the type of polycation, its charge density, the length of the polyanion, and the charge ratio of the coacervate components have to be optimized.

Drobot et al. were the first to show ribozyme activity in coacervates for a minimal version of the Hammerhead ribozyme (HH).^23^ Measurements in coacervate droplets and in isolated coacervate phase unequivocally proved that the ribozyme was active and able to cleave its substrate inside the coacervate. Apart from HH, other ribozymes (Figure 4.a) have been compartmentalized and shown to be active in coacervates: hairpin (HP) ribozyme displayed both cleavage^17^ and ligation activity,^20^ R3C ligase^50^ and a modified variant (E_L_-R3C) could concatenate RNA strands,^21^ and the Azoarcus ribozyme displayed autocatalytic assembly and ligation of its constituent fragments.^22^ In all cases, the ribozymes have been compartmentalized in complex coacervates formed by peptides,^20,21,50^ spermine^22^ or synthetic polymers^17,19,23^ in a highly charged microenvironment.

### 5.2 Concentrating Ribozymes and Substrates

Uptake of the ribozyme and its substrate(s) can be achieved by partitioning into preformed coacervates,^17,19,22,23^ or by condensation of the ribozyme and substrate with cationic peptides into RNA-based coacervates (Figure 4.b).^20,21,50^ The selected compartmentalization strategy has implications for the local concentration of ribozymes and their substrates: partitioning yields a local concentration of HH-ribozyme of 50 μM (*K*_p_ = 9600),^23^ whereas condensation is estimated to result in local oligonucleotide concentrations as high as several mM.^48,50^ However, despite the increased local concentration, not all studies reported a rate enhancement inside coacervates. CD measurements of HH revealed that the folding of the ribozyme was altered in pLys/CM-Dex coacervates, rendering it less active.^23^ The length, charge density and chemical nature of the other coacervate components all have a large effect on the observed ribozyme activity, as we will discuss below.

Cofactors can also be concentrated in coacervates by partitioning. Magnesium ions are particularly important for correct folding and substrate binding of many ribozymes. Although Mg^2+^ concentrations or partition coefficients are not reported in studies on ribozymes in coacervates, reports on other complex coacervates suggest that Mg^2+^ is likely concentrated.^48^ Moreover, experiments with HH and HP ribozyme at suboptimal Mg^2+^ concentrations indeed show that activity could be rescued in coacervates.^17^ This can be explained by effective charge screening by both Mg^2+^ which is locally concentrated, and the polycations inside the coacervate.

### 5.3 Tuning the Microenvironment to Enhance Ribozyme Activity

Coacervate components may interact with the ribozyme to alter its activity. Iglesias et al. showed that R3C ligase is active upon coacervation with cationic (RGG)_n_ heteropeptides, but inhibited when homopeptides (K_n_ or R_n_) were used.^50^ The reduced charge density of heteropeptides enables R3C activity in coacervates by promoting magnesium partitioning and RNA mobility (Figure 4.c). In coacervates with highly charged homopeptides, strong RNA binding may limit magnesium uptake while it results in RNA gelation through base pairing and kinetically trapped states.

Poudyal et al. also showed that the chemical nature of the polycation can influence ribozyme activity.^17^ Coacervates containing polycations with quaternary ammonium groups, such as PDAC, showed the highest HH and HP activity as they interact less strongly with ribozymes compared to primary amines or guanidinium groups (Figure 4.c). In a follow-up study, the authors also showed there is an optimal polyanion length for ribozyme activity: long polyanions prevented substrate partitioning, while short polyanions were unable to compete with HH, resulting in HH binding too strongly to the polycations and kinetic trapping or loss of its catalytic fold (Figure 4.c).^19^ In the same vain, excess polyanion could rescue HH activity for short polyanions, while it resulted in loss of activity for intermediate and long polyanions, because of substrate depletion from the coacervates.

The balance between ribozyme uptake and coacervate matrix interactions could also explain observations by Le Vay et al., who showed that substoichiometric amounts of polycation enhanced RNA ligation yields by a HP ribozyme (Figure 4.c).^20^ Excess polycation likely results in too strong binding of RNA and kinetic trapping, lowering ribozyme activity.

### 5.4 Pathway Selection

Finally, interaction between ribozymes and the coacervate matrix opens the possibility of pathway selection, as discussed in Section 4.3. Le Vay investigated the dual cleavage and ligation activity of HP,^20^ and observed a shift in the equilibrium from cleavage in solution to ligation in coacervates. We note that this effect was strongest for peptide-RNA coaggregates rather than coacervates. In another study, they found that coacervates could also suppress the formation of circular concatenation side products of the E_L_-R3C ligase (Figure 4.a),^21^ possibly resulting from a decreased RNA mobility, increased RNA chain stiffness or reduced product release. Interestingly, this enabled the formation of long RNA concatemers inside coacervates that alter the local environment in which they are produced: the coacervates became gel-like and resisted coalescence and wetting, which may be interpreted as a fitness advantage when these coacervates are used to create populations of protocells.^21^

## 6 Conclusions and Outlook

In summary, coacervates can accelerate prebiotic and ribozyme reactions through a multitude of mechanisms, which will usually act in concert. This makes it difficult to experimentally determine to what extent different factors contribute to rate acceleration. However, in most studies, only the partitioning of reactants is determined. Given that coacervates also play a role as membraneless organelles in living cells, there is considerable impetus for better characterization of the physicochemical properties of the coacervates in which reactions take place. This will help to gain insight into how the factors discussed in Section 4.2 influence reactions in coacervates and to assess their scope for prebiotic chemistry.

It is interesting to note that many of the mechanisms exploited by coacervates are also used in the enzyme active site. They similarly have an apolar local environment, different local p*K*_a_ of catalytic residues, accumulation of metal ion catalysts and lower water activity. Considering that many coacervates have been formed from peptide components, we close with a provocative thought that coacervates could perhaps have been evolutionary precursors of enzymes and might have even aided their emergence. Coacervates could have taken on rudimentary catalytic functions as “catalytic generalists” in an era before enzymes and could have paved the way for peptides to acquire defined active sites and evolve into enzymes as “catalytic specialists”. In such a scenario, coacervates did not necessarily have to be protocellular compartments that localized all reactions required for proto-life, but could have also aided the emergence of life by catalyzing specific reactions in the prebiotic soup as “proto-enzymes”.
